# Potentials of single‐cell biology in identification and validation of disease biomarkers

**DOI:** 10.1111/jcmm.12868

**Published:** 2016-04-26

**Authors:** Furong Niu, Diane C. Wang, Jiapei Lu, Wei Wu, Xiangdong Wang

**Affiliations:** ^1^ Huzhou Central HospitalHuzhouZhejiang ProvinceChina; ^2^ Department of Pulmonary MedicineThe First affiliated HospitalWenzhou Medical UniversityWenzhouChina

**Keywords:** single‐cell biology, mechanical phenotypes, gene sequencing, heterogeneity, genome function

## Abstract

Single‐cell biology is considered a new approach to identify and validate disease‐specific biomarkers. However, the concern raised by clinicians is how to apply single‐cell measurements for clinical practice, translate the message of single‐cell systems biology into clinical phenotype or explain alterations of single‐cell gene sequencing and function in patient response to therapies. This study is to address the importance and necessity of single‐cell gene sequencing in the identification and development of disease‐specific biomarkers, the definition and significance of single‐cell biology and single‐cell systems biology in the understanding of single‐cell full picture, the development and establishment of whole‐cell models in the validation of targeted biological function and the figure and meaning of single‐molecule imaging in single cell to trace intra‐single‐cell molecule expression, signal, interaction and location. We headline the important role of single‐cell biology in the discovery and development of disease‐specific biomarkers with a special emphasis on understanding single‐cell biological functions, *e.g*. mechanical phenotypes, single‐cell biology, heterogeneity and organization of genome function. We have reason to believe that such multi‐dimensional, multi‐layer, multi‐crossing and stereoscopic single‐cell biology definitely benefits the discovery and development of disease‐specific biomarkers.

## Introduction

A single cell plays an important and unique role in organ/tissue structures and functions where cell–cell communication is well accepted as an important mechanism for information exchange promoting cell survival and differentiation *via* cell secretome including soluble factors and exosome‐like vesicles to control population density and biological function. A single‐cell polarity can decide cell self‐function, cell communication and proliferation, of which alterations can induce the development of carcinogenesis and cell over‐proliferation. Key gene mutations in a cell can regulate intracellular signal pathways, influence intercellular communication and change cell biological function. With a growing understanding, the single‐cell biology is recently re‐emphasized and especially explored by sequencing a single‐cell DNA and RNA, defining epigenetics, constructing haploid and diploid maps of genome three‐dimensional architectures and improving single‐cell research methods. Furthermore, the single‐cell systems biology is a new approach to figure out the role of a single cell in organ function and pathogenesis of the disease.

**Figure 1:**
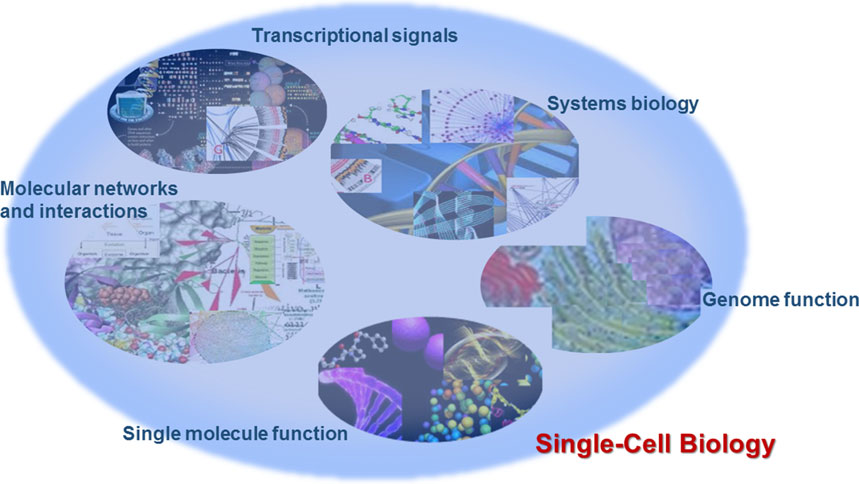
The complexity of single‐cell biology consists of single‐molecule function, molecular networks and interaction, transcriptional signals, systems biology and genome function.

Single‐cell biology is considered a new approach to identify and validate disease‐specific biomarkers. However, the primary concern raised by clinicians is how to apply single‐cell measurements for clinical practice, translate the message of single‐cell systems biology into clinical phenotype or explain alterations of single‐cell gene sequencing and function in patient response to therapies. This study is to address the importance and necessity of single‐cell gene sequencing in the identification and development of disease‐specific biomarkers, the definition and significance of single‐cell biology and single‐cell systems biology in the understanding of single‐cell full picture, the development and establishment of whole‐cell models in the validation of targeted biological function and the figure and meaning of single‐molecule imaging in single‐cell to trace intra‐single‐cell molecule expression, signal, interaction and location (Fig. 1). We headline the important role of single‐cell biology in the discovery and development of disease‐specific biomarkers with a special emphasis on understanding single‐cell biological functions, *e.g*. mechanical phenotypes, single‐cell biology, heterogeneity, and organization of genome function. We have reason to believe that such multi‐dimensional, multi‐layer, multi‐crossing and stereoscopic single‐cell biology definitely benefits the discovery and development of disease‐specific biomarkers.

## Application of single‐cell sequencing

Recently, single‐cell DNA or RNA sequencing has been used to investigate the genome of individual cells to demonstrate somatic mutations and clonal dynamics, define the cell‐type composition of the tissue and map the heterogeneity of cells in an area of tumour. To power the single‐cell sequencing and cover the variation of measurements, standardizations and guidelines of single‐cell study design, methodology, data analysis and interpretation become more important and urgent in practice 1. Kolodziejczyk *et al*. recently over‐viewed methodologies of single‐cell transcriptome sequencing and gave great efforts to suggest standardized protocols of single‐cell isolation, RNA sequencing and quality controls 2. Single‐cell sequencing becomes more and more important in single‐cell biology to understand development, immunology, neurobiology, cancer, gene regulation and epigenetics. It is questioned whether single‐cell sequencing can indicate or reflect disease mechanism, stage, severity or heterogeneity. One of the most challenging issues in single‐cell sequencing is to define and avoid variations in sampling, handling, reverse transcription, cDNA amplification or sequencing library preparation and differentiate the heterogeneity among patients, tumours, regions, cells and subpopulations. Another challenge is that the duration of measurements and analysis in single‐cell sequencing is too long to catch up with the needs of clinical monitoring and therapy.

**Figure 2:**
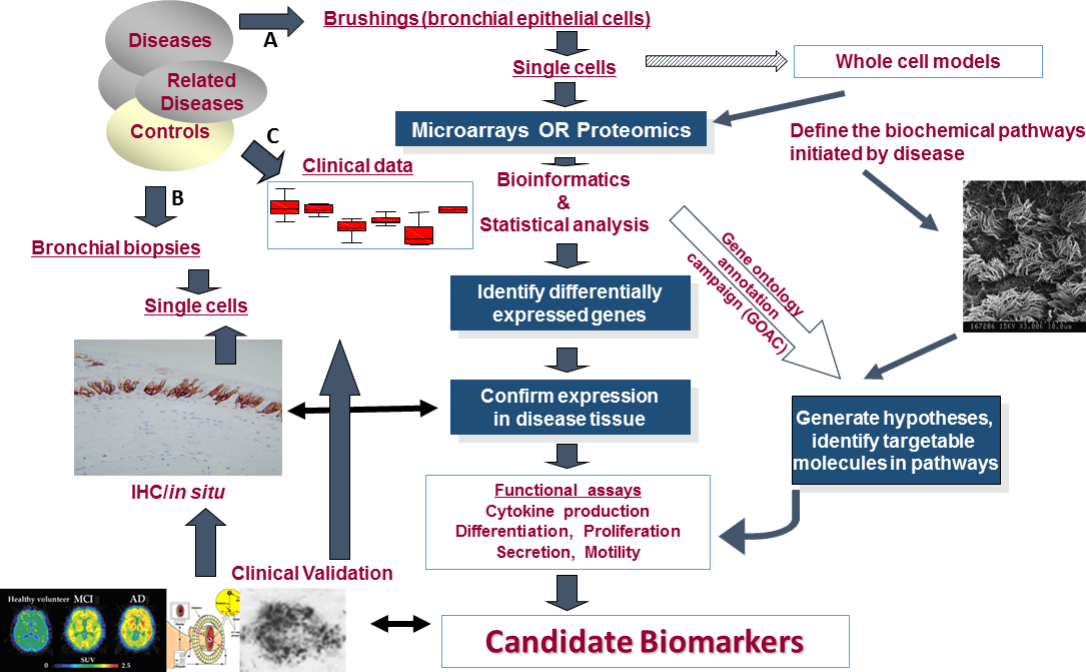
A workflow of clinical example to investigate single‐cell biology for the identification and validation of disease‐specific biomarkers. For example, bronchial single epithelial cells are isolated and purified by brushing the bronchial surface (**A**) or/and taking biopsies from pathological foci (**B**) of patients, *e.g*. with airway diseases after the collection of clinical information on patient phenotypes, biochemical measurements, molecular imaging, therapies and responses (**C**). Those single cells are applied for the measurement of gene expression, sequencing, epigenetics and function, and proteomic profiling, by integrating with the validation of intact single‐cell culture and function. Selected candidate biomarkers are furthermore validated and investigated in clinical systems, *e.g*. patient information, imaging, pathology and organ function.

On the other hand, it should be clarified that single‐cell RNA sequencing is mainly used to detect the gene expression and expressed mutation profiling, single‐nucleotide variation and circular RNA, while single‐cell DNA sequencing is used to identify somatic driver mutations, cell origins or heterogeneity in cell growth, drug resistance and metastasis. Single‐cell RNAs isolated from patient‐derived xenograft lung adenocarcinoma were sequenced for gene expression profiling and expressed mutation profiling, to identify a tumour cell subgroup associated with anti‐cancer drug resistance 3. It indicates that single‐cell transcriptome sequencing may also be applied for the optimization of clinical anti‐cancer strategies by integrating tumour‐specific single‐nucleotide variations, mutant expression and risk scores representing expression of lung adenocarcinoma‐prognostic genes, with tumour classifications (Fig. 2). We should be alarmed by the fact that data from single‐cell RNA‐sequencing measurements have to consciously be analysed to explain cell transcriptome function and biological variability to represent dynamic functions, rather than solely molecular noise 4.

## Significance of single‐cell systems biology

Single‐cell systems biology as part of single‐cell biology is a holistic approach to biomedical investigation of single cells, integrating computational science, mathematical modelling and high‐throughput technologies with biological function and organization in the cell. Nurse and Hayles called special attention from cell biologists on cell systems biology and headlined the necessity and importance of applying methods of systems biology more comprehensively to cell biology 5. Single‐cell biological function should be investigated and understood at different levels of biological organizations and systems, *e.g*. subcellular organelles, signal pathways or differentiations. Single‐cell systems biology also plays an important role in the ecology and evolution of the cell and organ, especially to understand how a cancer cell comes and differentiates to become more aggressive or metastatic or how an inflammatory cell immigrates and over‐activates to contribute to the formation of inflammatory microenvironment. Deep analysis of gene and protein expression profiles, metabolites or networks together with genome‐wide gene deletion collections and methods for saturation forward genetics can identify most genes in the genome involved in a cell function or process in physiological and pathophysiological conditions. It is exciting that single‐cell biology may be a critical approach to identify and validate disease‐specific biomarkers.

Single‐cell systems biology is a cell biology‐based interdisciplinary field of study on complex interactions within biological systems, to model and discover emergent properties involved with metabolic networks or cell signalling networks. Single‐cell systems biology is also a part of systems biology which covers the computational and mathematical modelling of more complex biological systems. Systems biology is the study of systems of biological components, which may be molecules, cells, organisms or entire species. Single‐cell systems biology is used for identification and validation of disease‐specific biomarkers at a system‐level understanding of living cell involvement by integrating synthetic biology and metabolic engineering with medicine, including single‐cell system structure, mechanism and dynamics of biological networks. Its system identification, analysis, control and design methods can help understanding of cell systems mechanisms to optimize cell biological function and define potential therapeutic targets in disease treatment at different levels of biological networks, dynamic networks or functional networks. The modification and construction of biological networks can provide system design principles and simulations for synthetic biology designs and systems metabolic engineering. It would be more important to develop single‐cell systems biology, systems synthetic biology and systems metabolic engineering 6.

## Potentials of whole‐cell models

Can the structure–function relationship of the single cells be detected on a human organ‐wide scale to understand the pathogenesis and pathology of the disease? Karr *et al*. reported a whole‐cell computational model of the life cycle of the human pathogen *Mycoplasma genitalium* to understand its molecular components, their interactions and complex phenotypes 7. Whole‐cell models are proposed as an efficient and integrative approach to describe or predict the process of single‐cell functions, a model to combine mathematics and simulate fundamentally different cellular processes and experimental measurements. Whole‐cell modelling has the potential to address annotated gene functions in single cells on the basis of a broad range of data, *in vivo* rates of protein–DNA association or an inverse relationship between the durations of DNA replication initiation and replication rates. The whole‐cell modelling is a computational and mathematical system or tool for translating genotype into phenotype to understand gene and cell function and to enable intelligent medicine and precision medicine. Whole‐cell modelling describes unobserved cellular behaviours or undetected kinetic parameters and biological functions from biological cell models which are an existed biology system and a model for biological responses. There is no doubt that such whole‐cell models can provide insights into identification and validation of disease‐specific biomarkers and knowledge to facilitate biological discovery of targets.

Whole‐cell models were confirmed to represent all cellular components at the molecular level and have the potential to predict phenotype from genotype. Yang *et al*. recently developed a new approach to facilitate fast, whole‐organ and whole‐body clearing with a direct delivery of clarifying agents *via* the circulation or the fluid route, including a quicker passive lipid extraction, Refractive Index Matching Solution and delivery of whole organ and body clearing and labelling 8. Such method could phenotype and describe single‐cell morphology, subcellular components and cell networks in an intact organ, although there is still need to be translated into human tissues and organs. It means that targeted single cells may potentially be microdissected for gene sequencing and epigenetic analysis to furthermore understand single‐cell morphology‐ and connection‐associated gene phenotypes and trace single‐molecule metabolisms within single cells. To characterize unknown parameters in whole‐cell models, a better and smarter strategy should include the power from the unique temporal and population structure of data, well‐developed simulation engines, distributed optimization algorithms, visualizations of differences among model simulations and new high‐throughput experimental technologies to define single‐cell variation and temporal dynamics 9.

However, there is an urgent need to understand more about how whole‐cell models can act as predictive tools with synthetic biology in single‐cell biology, engineer synthetic gene circuits in human single cells or be related with experimental trial‐and‐error on a cell. It is also questioned if whole single‐cell models would be able to reliably predict bio‐behaviours of synthetic circuits in the cell, reflect isolated circuits and complex cellular processes, or demonstrate dynamics and networks of the synthetic circuits. To use human whole single‐cell models to represent cell complex physiology and pathophysiology, there is a need for large amount of databases, analyses of simulations and software frameworks. For example, WholeCellSimDB was developed as a database for organizing whole‐cell simulations of a whole‐cell model of *M. genitalium* and three *Escherichia coli* models to search simulation metadata and share simulations with the broader research community 10. It was claimed to efficiently store, retrieve, search, browse, plot and export simulations, Web‐based visualizations, advanced analyses and functions, to advance basic biological science and bioengineering. It is important to design synthetic circuits and networks on the basis of human components, add genes into the host genome, correlate between codon usage and gene expression and establish human whole‐cell models. The implementation of a synthetic Goodwin oscillator in the whole‐cell model was suggested as an updated software framework and foundation for the whole‐cell models with synthetic gene circuits 11. The concept of whole‐cell system has been developed for multi‐applications, *e.g*. target mechanism‐based whole‐cell screening for validation of drug specificity and efficiency, quantitative whole‐cell proteome fingerprints for human monocyte subpopulations or living whole‐cell bioreporters serve as environmental biosentinels. Although whole‐cell models make a dramatic impact on single‐cell systems biology, bioengineering and medicine, a number of aspects should be further considered and developed, including experimental interrogation and data curation, model building and integration, accelerated computation and model validation, analysis and visualization and collaboration and community development 12.

## Imaging single molecules in single cells

Detection of single molecules in single cells with fluorescence *in situ* hybridization and digital imaging microscopy provides insights into dynamic alterations of intracellular transcription, sequencing, signalling, protein profile, metabolism and networks. Oligodeoxynucleotide probes are used to monitor synchronous and cyclical transcription from single genes, rates of transcription initiation and termination and mRNA processing in a single cell 13. It is possible to detect mRNA, DNA or protein profiles with single‐molecule sensitivity, map chromosome structure and protein–DNA or protein–protein interactions within a cell. Lubeck and Cai integrated multi‐transcriptional mRNA high‐solution imaging, computational labelling and bioinformatics to illuminate single‐cell systems biology 14. Chen *et al*. recently developed a single‐molecule imaging approach using multiplexed error‐robust fluorescence *in situ* hybridization to measure the copy numbers and spatial localizations of thousands of RNA species in single cells 15. Such revolutionary breakthrough with error‐robust encoding schemes to combat single‐molecule labelling and detection errors could demonstrate the imaging of 100–1000 unique RNA species, ~10^4^–10^6^ pairs of genes, gene regulatory networks, functions for many unannotated genes and properties of the encoded proteins. It opens new potential to study single‐cell genome medicine, measure gene expression and mutation and monitor gene‐editing precision and location in diseases. One of the most important advantages from such methods is being able to visualize single‐molecule processing, transport pathways, regulatory networks or signal interactions in a high‐throughput pattern, in a definite location and in a biological function 16. It is also a new approach to identify and validate disease‐specific biomarkers or therapeutic targets in a single‐molecule level within a single cell, by integrating clinical phenotypes and information (Fig. 2).

## Single‐cell biological functions

### Mechanical phenotypes

Mechanical phenotypes of single‐cell are suggested as a sensitive marker of cellular responses to pathophysiological changes. Cell stiffness and stiffness sensing as one of mechanical phenotypes play an important role in cell interactions, proliferations and differentiations. Altered cell stiffness was found to contribute to the development of diseases, *e.g*. vascular diseases, hypertension or cancer. Reduction in cancer‐cell elasticity and stiffness‐sensing ability could cause the loss of cancer cells to response to microenvironmental changes and was suggested as important biomarkers of a cancer‐cell phenotype, mechanosensation or mechanotransduction, probably through Cav1‐upregulated RhoA activity and Y397FAK phosphorylation 17. Mietke *et al*. used real‐time deformability cytometry to measure model spheres of known elasticity and biological cells and cell mechanical parameters with the quantification, allowing for mechanical phenotyping based on single‐cell deformability 18. However, there are a number of influencing factors in measurement of cell stiffness, *e.g*. device systems, analytic methods, channel conditions or cell properties. It also lacks the knowledge of cell stillness in complex conditions of diseases.

### Single‐cell biology

A number of single‐cell–based disciplines are generated with an increasing knowledge of single‐cell functions, *e.g*. single‐cell immunology, single‐cell biology, single‐cell systems biology, single‐cell pharmacology, single‐cell toxicology or single‐cell omics. For example, the variability is a process by which an immune single cell of the same population has different responses to a pathogen with slight, modified, or mutated changes, whereas the heterogeneity is a property of which different single cells of the same population respond to the same pathogen differently. Avraham *et al*. showed that the macrophage population did have heterogeneous infection outcome under the condition that the content of inflammatory mediators varied among single macrophages; whereas the ‘same’ macrophages had different responses to variable *Salmonella* population 19. This may be a new molecular mechanism to explain the heterogeneity of immune responses to pathogen, to therapies, to environmental changes or even during the disease. The heterogeneity of single immune cells may result from inherited processes, environmental factors, living conditions or pathogens, although the exact mechanism remains unclear.

### Heterogeneity

**Figure 3:**
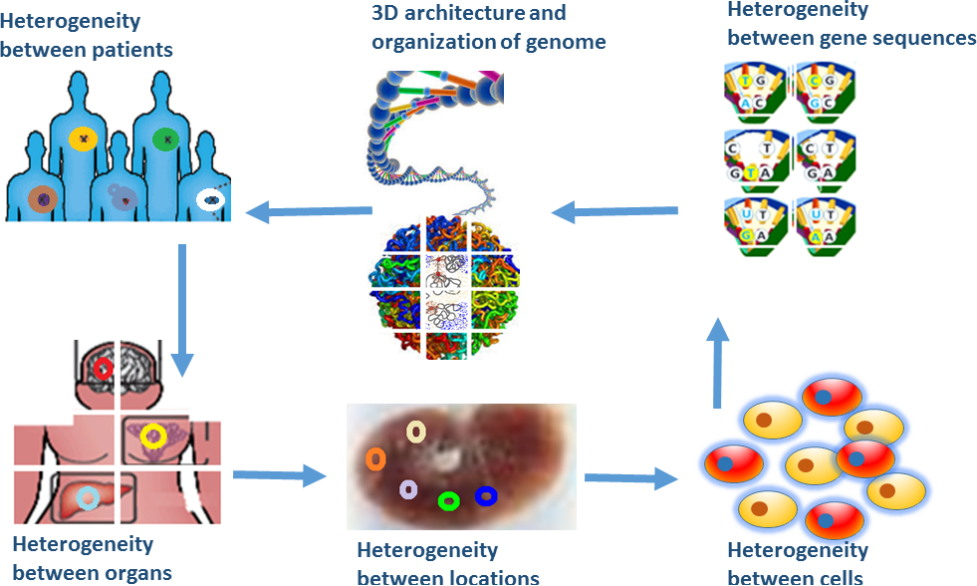
Presence of heterogeneity or variability among species, populations, patients, inter‐organs/tissues, intra‐tumour locations, cells within a location, gene mutations and variations, and 3D architecture and organization of genome function.

There is growing evidence to show that there is heterogeneity or variability among species, populations, patients, inter‐organs/tissues, intra‐tumour locations or cells within a location (Fig. 3). The cell heterogeneity can be detected by gene sequencing, proteome profiling, biological functioning or cytoplasmic transcript abundance. It appears that heterogeneity or variability exists in the body wherever, whenever, whatever and whichever. Battich *et al*. investigated variability in cytoplasmic transcript where abundance is measured by multi‐level transcript homeostasis in single cells with image‐based transcriptomics in millions of single human cells and demonstrated that variability was in unexpectedly large amounts. Variability in most genes can be minimalized and predicted with multivariate models of the phenotypic state and population context of single cells 20. There is a comprehensive regulatory system in control of such variability among single cells, consisted of a large number of transcripts reserved and transported between the nucleus and the cytoplasm through the nuclear compartmentalization to buffer stochastic transcriptional fluctuations in gene expression. Such single‐cell heterogeneity or variability may provide more detailed information to understand molecular mechanism of disease, which may be more complex or difficult than expected. On the other hand, more precision methodologies are urgently needed to clarify and filter the transcriptional noise, gene/protein interaction and networks, or measurement sensitivity.

### Genome functions

Genome structure and function dominate the cell function and phenotype in post‐genome era, especially after the rapid development of single‐cell biology. Misteli clearly states that we should pay more attention to cellular organization of genome function 21, in addition to gene sequencing, as the spatial arrangement of the chromatin fibre and the genome can affect the function of DNA. We have to remember that gene sequencing is only part of genome function and that gene mutation plays partial role in the pathogenesis of disease. It is a misunderstanding and misleading that the measurement and understanding of gene sequencing *per se* are defined as ‘Precision Medicine’, or that the correction of one mutation can cure a cancer. Genome functions are characterized by a number of factors, *e.g*. long‐distance chromatin or positioning of genomic loci closely associated with nuclear functions, *e.g*. transcription, replication, DNA repair and chromosome. Positioning of genomic loci can be influenced by chromatin remodelers, histone modifiers, nuclear envelope and pore proteins, or elements of the replication and after replication chromatin reassembly machinery. Misteli group recently identified a number of cellular factors for proper positioning of a set of functionally diverse genomic loci and mapped the spatial location of genome regions at large scale with a high‐precision, high‐throughput, automated fluorescent *in situ* hybridization imaging pipeline 22. Chromosome repositioning can influence gene activity and regulation, genome stability or related molecular mechanisms of cellular pathways, leading to alterations of cell function.

The spatial organization of the human genome plays an important role in the transcriptional control of genes. The self‐enforcing feedback and interaction between activity and spatial organization of the genome form a self‐organizing and self‐perpetuating system to regulate genome function. Rao *et al*. used *in situ* Hi‐C combining DNA proximity ligation with high‐throughput sequencing in a genome‐wide fashion, to probe the 3D architecture of genomes at kilobase resolution and demonstrate principles of chromatin looping 23. Results from this particular study painted all genomic loci, demonstrated the partitioning of the genome into numerous domains and loops across the genome and figured out DNA–DNA proximity ligation in intact nuclei. In addition, dynamics of genome functioning and structuring with multi‐factors might be another dimension to be considered seriously and form a 4D genome with temporal information collected together with high‐resolution spatial information both along the chromatin fibre and in 3D interaction space. It is critical to not only identify the heterogeneity of single‐cell sequencing, but also integrate with molecule imaging and function; not only measure the expression of genes and proteins, but also define organization of genome function; not only observe molecular imaging, but also figure out intact cell function; not only investigate single‐cell profiles in cancer, but also in other diseases like inflammation 24.

### Biology‐specific biomarkers

Biomarkers have attracted more and more attentions and categorized into disease biomarkers to monitor disease stage, duration, severity and response to therapy 25, 26, 27, function biomarkers to describe the interaction between molecules, networks and dynamic networks 28, 29, 30, prognostic biomarkers to predict the outcome of patients after the treatment 31, 32, 33, genetic biomarkers to reflect alterations of gene mutations and function 34, 35 or drug biomarkers to show drug efficacy, metabolism, pharmacodynamics, absorption and toxicology 36, 37, 38. One of critical factors in the performance of precision medicine was proposed as the identification and development of biology‐specific biomarkers to monitor each process and form of gene mutation and vulnerability during DNA damage and repair 39. We need biology‐specific biomarkers to monitor the function and activity of single cells independently and interactively. With the development of advanced biotechnologies 40, 41, 42, 43, a large number of biomarkers and targets will be discovered and validated during the investigation of single‐cell biology and systems biology. One of challenges is to identify the biology biomarkers for the precision of whole‐cell modelling, reliability of data from bioengineering and databases and genome function.

In conclusion, we call special attention on the important role of single‐cell biology in discovery and development of disease‐specific biomarkers, by further understanding proper application of single‐cell gene sequencing, systems biology, whole‐cell models, single‐molecule imaging and single‐cell biological functions, *e.g*. mechanical phenotypes, single‐cell biology, heterogeneity and organization of genome function. We have reason to believe that such multi‐dimensional, multi‐layer, multi‐crossing, and stereoscopic single‐cell biology definitely benefits the discovery and development of disease‐specific biomarkers.

## Competing interest

The authors declare no competing of interests.
